# Self-reported knowledge, attitudes, practices and barriers in use of evidence-based medicine among resident physicians in Kenya: a mixed methods study

**DOI:** 10.1186/s12909-021-02974-4

**Published:** 2021-10-23

**Authors:** Megha B. Unadkat, Caroline K. Mbuba, Anthony K. Ngugi, Dorothy Kamya

**Affiliations:** Aga Khan University Hospital, Nairobi (AKUHN), Nairobi, Kenya

**Keywords:** Evidence Based Medicine, Evidence Based Practice, Postgraduate Medical Education, Mixed methods, Knowledge, Attitude, Practice, Barriers

## Abstract

**Background:**

Evidence based medicine (EBM) helps clinicians to integrate latest research evidence into their daily clinical practice. There is a need for all healthcare professions to adopt it in order to provide safe and most cost-effective care. Postgraduate doctors are at the frontline of healthcare delivery and all medical institutions should strive to produce practitioners of EBM. Studies have shown that physicians are still struggling to adapt to this paradigm shift in the practice of medicine but very few studies have been done in Sub Saharan Africa. This study explored the self-reported knowledge, attitudes, practices and barriers of evidence-based practice among resident physicians in a tertiary teaching hospital.

**Methods:**

A mixed methods cross-sectional study that used convergent parallel design was conducted. The quantitative arm was conducted among all residents enrolled in the Master of Medicine programme at Aga Khan University Hospital Nairobi (AKUHN). It included an online survey exploring self-reported knowledge, attitudes, practices and barriers of EBM among all residents. Simultaneously, semi-structured In-Depth Interviews were carried out among 18 purposefully selected residents in order to explore the same themes in more depth.

**Results:**

One hundred and one residents (99%) responded to the survey. The mean scores for self-reported knowledge, attitude and practice of EBM among residents were 73.88, 66.96 and 63.19% respectively, which were generally higher than in comparable studies. There was a significant association between year of residency and practice of EBM. The most common barriers faced by residents were lack of time, lack of EBM skills and patients’ unawareness about EBM.

From the qualitative study, residents demonstrated good knowledge and support of EBM but practice remained relatively poor. Barriers to EBM were characterized by lack of motivation, time, skills and resources, patient overload and fear of challenging consultants.

**Conclusion:**

There was good understanding and support of EBM among residents at AKUHN, though challenges were experienced in regards to practice of EBM because of lack of time and skills. Therefore resources should be allocated towards integrating EBM into undergraduate medical curricula to cultivate critical thinking skills at an early stage before transition into residency.

**Supplementary Information:**

The online version contains supplementary material available at 10.1186/s12909-021-02974-4.

## Background

Evidence-Based Medicine (EBM) is defined as “the conscientious, explicit and judicious use of the best evidence in making decisions about the care of patients” [1]. Practice of EBM provides a paradigm shift in the way clinical services are offered and medical education undertaken.

There are five main steps to practice EBM which include developing a well articulated clinical research question, searching for the research information, critically appraising the research evidence acquired, determining applicability of the evidence to the patient and evaluating the overall performance [2]. The challenge faced by most physicians is acquisition of skills on how to carry out efficient searches and systematically critique the literature for evidence [3].

Despite a 20 years-history of EBM and increased availability of EBM resources, many practicing clinicians, especially in Low and Middle Income Countries (LMIC), are still not exposed to this global trend and critical appraisal skills are still not part of formal training [4]. Medical residents are usually at the frontline of healthcare provision in teaching hospitals, in addition to their responsibility of teaching their juniors, and therefore they play a key role in the integration of EBM into daily practice [5].

The high disease burden in Africa associated with poor health infrastructure and limited resources raises the need for cost-effective approaches to integrating best available research evidence into policy-making and clinical practice. Although important strides have been made to improve research productivity in Africa, there is limited effort to promote use of evidence overall and practice of EBM in particular, at both undergraduate and postgraduate levels [6]. For example, up to 70% of the post-graduate doctors use textbooks as main source of information in Africa and major barriers to the use of EBM were identified as unawareness of the EBM resources, power interruptions and lack of computing facilities [7].

Few rigorous studies have been conducted in LMIC as well as in high-income countries to explore the knowledge, attitudes and practices of EBM among residents. In a study conducted in Sudan, only 10% of the residents used EBM for more than 50% of their practice [8]. In Saga University Japan, again only slightly over half of the residents understood EBM terminology, but many considered EBM practice to improve patient care and nearly all of them believed that EBM also assisted in clinical decision making [5]. Similarly in Iran, only half of the residents were familiar with EBM concepts but a majority of them were positive towards the idea of practicing EBM [9].

A systematic review of 28 studies conducted in Iran studied the barriers, knowledge and attitude towards EBM among different target groups including nurses, physicians, residents, dental students as well as medical students [10]. This systematic review illustrated that the degree of familiarity with EBM was low and that textbooks remained the key source of information, with only 50% practicing EBM. In contrast, a study conducted in Ethiopia among interns and residents, showed that 75.5% had heard about EBM and widely used EBM resources during clinical decision making included UpToDate, Google and PUBMED [11].

Barriers to the practice of EBM appear to fall into three different categories: personal, organizational and patient-related barriers. Personal barriers include lack of familiarity and use of EBM resources [5], discomfort going beyond trusted clinical routines [12], lack of experience in forming research questions and hierarchical dependence on senior staff. Other personal barriers include senior physicians’ resistance to change their practice and lack of access to EBM training opportunities [13]. Organizational related barriers include patients overload, lack of libraries and information technology resources in the hospitals and lack of time [8, 10].

There is very limited research into the awareness, perceptions and use of EBM in Sub Saharan Africa (SSA), especially in East Africa. The overall aim of this study was to evaluate the knowledge, attitudes and practices of EBM among resident physicians at Aga Khan University Hospital Nairobi (AKUHN) and perceived barriers to practicing EBM during patient care.

## Methods

### Design

A mixed methods cross-sectional study that used convergent parallel design was conducted among residents in AKUHN. The purpose of the combined approach was to provide a better and a more complete understanding of the research problem as well as side by side comparison of findings from both studies. The qualitative findings provided more information to support or explain the quantitative findings. The In-Depth Interviews (IDIs) allowed participants to expand on their thoughts and personal experiences as well as for the interviewer to probe further on certain interesting points raised by participants. Overall, this method provided a more complete picture of the situation.

### Study setting

The study was carried out at AKUHN, which is a private not-for-profit tertiary level teaching hospital established in 1958. As a private institution, it serves mostly patients from the middle to high socioeconomic class; mostly people with medical insurance.

The Post Graduate Medical Education (PGME) programme in AKUHN currently offers training programmes in the following nine specialties: Anaesthesiology, Anatomic pathology, Clinical pathology, Family medicine, General surgery, Internal medicine, Imaging and diagnostic radiology, Obstetrics and gynaecology and Paediatrics & child health.

Each programme lasts 4 years leading to a Master of Medicine (MMed) degree. There were 102 residents enrolled in the programme during the period of this study. EBM is one of the ‘common’ courses offered in AKUHN in part one of the MMed programme. A common course is a mandatory, crosscutting training course offered to all residents, regardless of training specialty. The course is aimed at improving residents’ skills and knowledge about EBM [14, 15]. The course is conducted by the library and PGME personnel and covers key aspects of EBM: introduction to available E-learning resources, critical appraisal of the literature and Research Methods. These skills are further embedded in departmental weekly journal club meetings and faculty academic rounds.

### Study participants

Participants in the quantitative phase were students from all nine MMed degree programmes, but in different stages of their PGME residency. First and second year residents are “Junior” and third and fourth year residents are “Senior”. These senior residents having been in the programme longer had more exposure and opportunities to implement EBM.

Purposive and quota sampling methods were used to identify two key informants from each of the nine departments for the IDIs [16, 17]. This sampling method was chosen to reflect diversity with respect to different departments, stage of training, gender and willingness to participate in IDIs. Consequently, 18 residents were selected, nine junior and nine senior.

### Data collection

#### Quantitative phase

Quantitative tool - Data were collected using an online self-administered 5-point Likert scale questionnaire sent via Survey Monkey®. It was comprised of five sections consisting 64 items (Additional file 1).

The questions were adapted from a validated questionnaire, used in a study conducted in Saga University teaching hospital [5] and from other studies [11, 18-20]. The self-administered questionnaire was adapted to suit the local set-up, adding resources and barriers specific to the AKUHN library/system. It was revised slightly after feedback from a pilot study done on ten alumni to check for clarity and flow.

Recruitment and data collection: All 102 residents were informed about the study before questionnaires were sent out. The principal investigator (MU) visited all nine departments during academic sessions and explained the study and invited residents to participate in an online survey. Following the online survey, purposive and quota sampling methods identified residents for IDIs.

Invitations to participate in the study were sent with the questionnaire to all 102 residents via Survey Monkey®. Reminders were sent weekly, then daily for non-responders. The survey was conducted between September–October 2017.

### Qualitative phase

Selected participants were contacted by telephone call to set a suitable date and time for the IDIs. Participants read a detailed information letter before they signed a written informed consent form. Qualitative data were collected using an interview guide, consisting of the following sections: knowledge, attitude, practice and barriers of EBM (Additional file 2). The principal investigator conducted the IDIs; probing respondents’ answers to generate richer data.

Interviews lasted between 30 to 40 min and were audio-recorded. They were conducted in a quiet private room to allow residents to feel comfortable and to avoid interruptions. The participants were interviewed between September–December 2017.

### Analysis

#### Quantitative

Quantitative data were analyzed using SPSS version 20. Frequencies of different socio-demographic strata (age, sex, department and year of study) were used to describe the study population. The total scores obtained by all residents on all the individual items on the Likert scale were computed for each variable of knowledge, attitude and practice, ensuring that the categories assigned to the negative statements (especially when assessing attitude) were scored by reversing the scale. For evaluation of the self- reported knowledge, attitudes and practices of EBM among residents; the total scores were divided by the highest possible scores to obtain the mean and Standard Deviation (SD) scores for each variable.

Tests of association such as simple linear regression were used to examine the relationship between socio-demographic variables (age, sex and year of study) and the knowledge, attitudes and practices towards EBM. All variables with a *p*-value of ≤0.25 in the univariate analyses were then included in the multiple linear regression model to identify their combined effect on the outcomes. *P*-value of < 0.05 was considered statistically significant. Parameter estimates: Linear regression coefficients, 95% Confidence Intervals (CI) and *p*-values were recorded for each explanatory variable.

#### Qualitative

Qualitative data were systematically analyzed using thematic framework analysis [21]. Audio-recorded interviews were transcribed verbatim into textual data. Familiarization of data occurred by reading and re-rereading each transcript thoroughly. Two members of the research team (MU & CK) independently coded and generated themes from the data. Both descriptive and In-Vivo codes were generated. Any disagreement on codes and emerging themes was resolved through discussion. This process served to maximize the rigor and credibility of the analysis. A picture of the whole data was then constructed by charting followed by mapping and interpretation of the findings.

## Results

One hundred and one residents (99%) responded to the survey. The socio-demographic characteristics of respondents are shown in Table 1.

**Table 1:** Socio-demographic characteristics of 101 study participants

Participants (***n*** = 101)		n (%)
Age	< 30 years	40 (39.6)
	≥ 30 years	61 (60.4)
**Sex**	Male	58 (57.4)
	Female	43 (42.6)
**Department**	Anaesthesiology	11 (10.9)
	Anatomic Pathology	11 (10.9)
	Clinical Pathology	7 (6.9)
	Family Medicine	7 (6.9)
	General Surgery	12 (11.9)
	Internal Medicine	14 (13.9)
	Imaging & Diagnostic Radiology	13 (12.9)
	Obstetrics & gynaecology	12 (11.9)
	Paediatrics & Child Health	14 (13.9)
**Year of study**	One	30 (29.7%)
	Two	26 (25.7%)
	Three	23 (22.8%)
	Four	22 (21.8%)

The mean scores for self-reported knowledge, attitude and practice of EBM among residents were 73.88% (SD 13.74), 66.96% (SD 6.54) and 63.19% (SD 8.43) respectively, as shown in Table 2.

**Table 2:** Mean scores for self-reported knowledge, attitude and practice of EBM at AKUHN

	Total score (n/N)	Mean score (%)	Standard Deviation
**Knowledge**	8698/11615	73.88	13.74
**Attitude**	6176/9090	66.96	6.54
**Practice**	2917/4545	63.19	8.43

### Self-reported knowledge about EBM

**Fig. 1:**
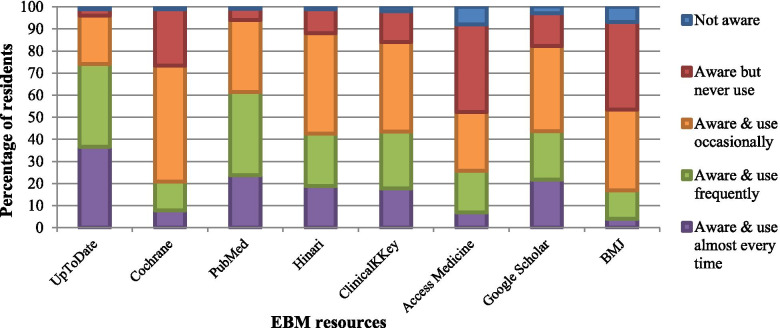
Familiarity with EBM resources among 101 residents at AKUHN

The most common EBM resources respondents were aware of and used most frequently were UpToDate (74.2%), PubMed (61.4%), Hinari (42.6%), Google Scholar (43.6%) and Clinical Key (43.5%), as shown in Fig. 1.

This is further illustrated in qualitative findings: *“When you’re not on call, doing daily work, then it’s easy you get to Hinari, UpToDate, PubMed directly …*. *it is very easy for us”. (R9).*

### Self-reported attitude towards EBM

**Fig. 2:**
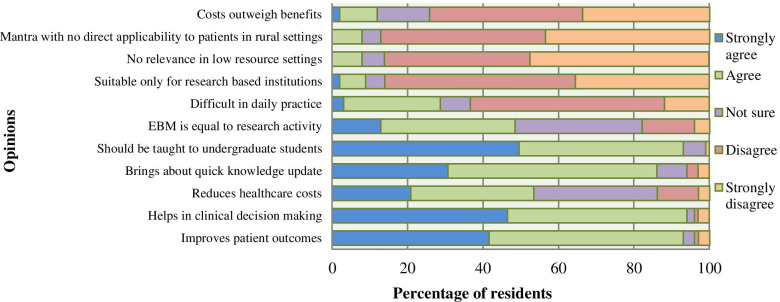
Opinions of 101 residents about the practice of EBM at AKUHN

More than 90% of the respondents agreed or strongly agreed that EBM improves patient outcomes, and that it helps in clinical decision-making and should be taught to undergraduate students. A majority (> 80%) of the residents also disagreed or strongly disagreed that EBM is a mantra with no direct applicability to patients in rural settings, that it had no relevance in low resource settings and that it is suitable only for research based institutions. Congruently, 74.3% of the residents either disagreed or strongly disagreed that the costs of EBM outweigh the benefits, indicating that EBM is cost-effective (Fig. 2).

The qualitative findings were similar; most respondents were convinced that EBM had significant impact on ensuring safer patient care: *“Before I came here (AKUHN), I used to follow my consultant, I didn’t know how to look for stuff myself. And I think we used to give substandard care to patients out there. (R16).*

Respondents believed that EBM was important in a number of ways, right from medical school to shaping future consultants. It was also vital as part of their residency as it made them competent doctors at an international level: *“EBM should be taught in undergraduate level because if you are taught by someone whose practicing style is the old way then you have a muted experience and you don’t know how to approach EBM. The sooner you learn, the better your approach”. (R2)*

*“It is important for residents in order to be at par with everyone and you can practice medicine anywhere in the world and follow international treatment protocols and work in internationally accredited hospitals”. (R13)*

### Self-reported practice of EBM

**Fig. 3:**
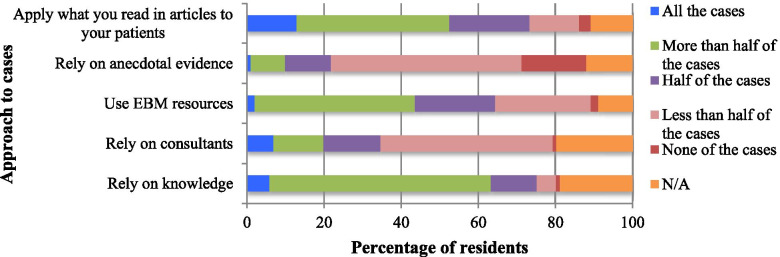
Self-reported practice of EBM among 101 residents during the previous month of working at AKUHN

Sixty five percent of the respondents said that they used EBM resources for half or more of the cases seen, 34.7% relied on consultants for half or more of the cases and 75.2% mostly relied on their knowledge to treat half or more of the cases (Fig. 3).

Despite a good understanding of EBM and of the facilitating factors, actual application of EBM in day-to-day practice was quite low. Most respondents indicated referring to EBM sources for only 20–30% of patients on a daily basis: *“Half the time you’re running around, checking up on results, doing procedures, so the time to sit down and start looking through data is impossible, 30% at most”. (R18).*

In the survey, more than 90% of the respondents identified encountering an uncommon case or for grand rounds or case discussions as the most common reasons for practicing EBM, a finding echoed in the interviews. Other common reasons identified for practicing EBM were: the patient not improving on current management, treating a new patient: *“Sometimes you don’t dig that much unless you see a complicated patient. So not for every patient”. (R17).*

#### Barriers to the practice of EBM

The barriers faced by respondents are categorized into personal barriers, organizational barriers and patient-related barriers as shown in Table 3.

**Table 3:** The mean scores for each of the barriers to EBM faced by AKUHN residents

Barriers	Total score (n/N)	Mean score	Standard Deviation
Lack of familiarity with EBM	267/101	2.64	1.18
EBM practice devalues clinical experience	194/101	1.92	0.74
Impracticality of EBM for everyday use	248/101	2.46	1.03
EBM removes the ‘art’ of medicine	200/101	1.98	0.77
EBM de-emphasizes history taking and physical examination skills	195/101	1.93	0.78
Lack of time to access EBM sources	338/101	3.35	1.18
Insufficiency of basic EBM skills	300/101	2.97	1.14
Skepticism over the quality of evidence	274/101	2.71	1.10
Patients’ unawareness about EBM and preference of traditional approach	286/101	2.83	1.09

The most frequently identified organizational barrier faced by respondents was lack of time to access EBM resources due to heavy workload, with mean score of 3.35 (SD 1.18). This was also reflected in the qualitative findings: *Number one barrier is large number of patients- so you do just bare minimum for each and just move on”. (R12).*

The barrier with the second highest mean was a personal barrier – “insufficiency of basic EBM skills” with a mean score of 2.97 (SD 1.14) (Table 3). Respondents described their struggle to practice EBM in their daily practice due to a lack of awareness and expertise in critical thinking. This they attribute to never having been taught to question or think broadly since undergraduate education: *“Searching takes up the most time because … people have to be taught technique for searching, there is a systematic way”. (R5)*

*“Kenyan education makes us just follow orders, we are not taught to think outside the box from school. So we continue doing things a certain way without asking questions”. (R5)*

Many respondents felt that “Patients’ unawareness about EBM and preferences of traditional approach” was also a barrier to practice of EBM with a mean score of 2.83 (SD 1.09) (Table 3).

Conversely, this was not expressed as a concern in the qualitative findings: patients were felt to have access to too much health information. Residents reported that motivated them to read more: *“It stops you from slacking so I take it as positive and motivates me to up my game”. (R13)* Most felt that access to health information by patients had a positive influence on their practice of EBM and acted as a facilitator instead of a barrier: *“An informed patient was a good patient”. (R15).*

### Relationship between self-reported knowledge, attitude, practice and barriers of EBM and socio-demographic factors – sex, age and year of residency

There were no significant variations in knowledge and attitudes towards EBM across the three participant characteristics (Table 4). However, the practice of EBM varied with the year of residency. Each unit increase in year led to a corresponding 1.48 increase in the mean score for practice of EBM (*p*-value = 0.01).

**Table 4:** Relationship between level of self-reported knowledge, attitude and practice and socio-demographic factors – sex, age and year of residency

		Linear Regression Coefficient (95% CI)	***P***-value	Multiple Linear Regression Coefficient (95% CI)	***P***-value
**Knowledge**	**Sex**	−3.50 (−8.97–1.98)	0.21	−3.16 (−8.62–2.31)	0.25
	**Age**	0.75 (−2.27–1.72)	0.13	0.53 (−0.52–1.59)	0.32
	**Year**	1.89 (−0.51–4.29)	0.12	1.26 (−1.34–3.87)	0.34
**Attitude**	**Sex**	1.50 (−1.11–4.10)	0.26		
	**Age**	− 0.16 (− 0.63–0.31)	0.50		
	**Year**	0.11 (− 1.04–1.27)	0.85		
**Practice**	**Sex**	1.10 (− 2.27–4.48)	0.52		
	**Age**	−1.03 (−4.21–2.16)	0.52		
	**Year**	1.13 (− 0.34–2.60)	0.13	1.48 (0.40–2.57)	0.01

## Discussion

### Self-reported knowledge about EBM

The overall mean score for EBM knowledge among residents at AKUHN was higher than in comparable studies done in other settings [9, 11]. The level of familiarity with EBM and its concepts among residents in this study is high, compared to residents in similar settings. This may be because of the introductory EBM course at AKUHN conducted during their orientation. AKUHN residents described the course as useful as it provided a strong foundation to build on. However, they felt it was too short and over-packed. Residents’ ready access to the online library and to activities at AKUHN such as journal clubs, surgical seminars and Continuing Medical Education may have further enhanced their understanding and familiarity of EBM. This contrasts to Sudan where no formal training in EBM resulted in gaps in knowledge, skills and awareness [8]. Most medical professionals in Sudan base their clinical judgments on their experience, consultations with colleagues and common sense [22].

Unlike studies done in high income countries like Canada, Norway and Israel in which physicians reported limited knowledge of EBM and its concepts [23-25], residents in the AKUHN study reported a good understanding of EBM. This is an interesting finding and may have several explanations. The studies done in higher income countries were done among physicians in both academic and community-based settings where participants were not necessarily in training during the time of the study. AKUHN is an academic medical setting and its residents are in specialist training which has a mandatory research component; this could have contributed to their higher level of EBM knowledge in comparison to the physicians in other studies. The possibility of inaccurate self-reporting also arises here; AKUHN residents may have felt reticent about admitting unfamiliarity with EBM. In view of the fact that they had received EBM training and had access to readily available EBM resources, they may have self-reported a higher level of familiarity with EBM than was true. Finally, these differences could be due to the use of different data collection tools and methods in the four studies and thus may not be directly comparable.

The lack of significant association between year of residency and knowledge of EBM is interesting and is echoed in the Japanese study [5]. Knowledge is a dynamic concept and some aspects may change over time but others may not. It is possible that the residents’ EBM knowledge in itself did not progress over the years but the way it was utilized and applied may have. All residents in this study acquired EBM knowledge early in their training and with time, availability of EBM resources and encouragement to apply EBM, senior residents probably advanced in their skills and practice of EBM more than in their knowledge of EBM.

AKUHN residents were more aware of and used: UpToDate, PubMed and Hinari than participants in the Japanese and Ethiopian studies who used the BMJ Clinical Evidence journal, UpToDate, Google and PubMed to guide their clinical practice [5, 11]. Studies in literature have shown that UpToDate has become a very popular tool among residents because it answers clinical questions at the point of care with speed and ease [2, 26]. The findings in this study confirm the popularity of UpToDate because it provides the most synthesized data in a logical manner, is easy to use and can be accessed from different personal devices through a subscription, which AKUHN offers to its residents [27]. AKUHN library data showed variable use of UpToDate across different specialties - paediatricians and internal physicians had the highest number of searches and anaesthetists the lowest. This finding could be due to the fact that UpToDate covers more content and topics from internal medicine and paediatrics compared to anaesthesiology or radiology. The finding that surgical and anaesthesiology residents find it difficult to look up evidence at point of care, which can be in the middle of a procedure, reflects their lower utilization of the resource.

### Self-reported attitude towards EBM

The mean score of attitude for residents at AKUHN was higher than reported in the systematic review of 28 studies done in Iran reflecting residents’ positive attitude towards EBM at AKUHN [10]. Their level of positivity for EBM is similar to that in studies done in Iran, Ethiopia and Sudan [9, 11, 22]. The positive attitude of AKUHN residents rooted in their perception of the EBM as being “cost-effective” for patients. Residents at AKUHN embrace the use of EBM, which is a paradigm shift in the practice and teaching of medicine in Kenya and worldwide [1]. Residents felt that EBM can only be practiced effectively in a resource rich setting. Most hospitals in Kenya may not enjoy the level of resourcing as the study site, making EBM a resource that can be afforded by a minority of medical practitioners in the local set-up.

In SSA, EBM is usually not a part of the Undergraduate Medical Education curriculum. A university of Lagos study showed a positive impact on the medical students following introduction of an EBM course. The graduates reported a significant enhancement in their knowledge and attitude towards EBM and acquisition of lifelong learning skills [28]. Congruently, the residents in this study strongly believed that EBM is a practice that should be instilled right from undergraduate to create better future EBM practitioners. Thus, if the skills of literature search and critical appraisal were taught right from medical school, then residents would find it easier to continue the practice into residency.

### Self-reported practice of EBM

At odds with the findings of relatively high knowledge of and positive attitude to EBM was the low level of EBM practice at AKUHN. This is similar to the figure in a study conducted in Sudan, which reported EBM practice level of less than 25% of the time. Moreover, in this study among AKUHN residents, EBM practice was variously rated as high in the quantitative but low in the qualitative portion of the study. This discrepancy may be related to the different methodological approaches, which permit different degrees of expansive responses. It may result from the barriers reported: lack of time, resources and skills. Indeed, the high usage of EBM reported in the quantitative survey could be due to self-reporting bias. Thus, residents could over-report their usage of EBM because of inaccurate recall of their practice or perhaps to portray themselves positively.

A statistically significant relationship was found between year of residency and the practice of EBM in this study; as the year of residency increased, the practice of EBM also increased. So, as residents progress in their training from junior to senior level, they become increasingly at ease with the practice of EBM, learning by doing. The graded responsibilities of senior residents as they assume an increased level of clinical responsibility require them to be able to research and critically appraise medical literature and apply the acquired information directly to patient care or share the knowledge with their junior colleagues.

### Barriers that hinder practice of EBM at AKUHN

A majority of the residents in AKUHN experienced a number of barriers when implementing EBM, which were categorized into organizational, personal and patient related barriers. The most common barrier faced by residents was lack of time to access EBM resources, an organizational barrier. Similarly, lack of time and heavy workload were the most common challenges faced by doctors in studies conducted in Japan and Sudan [5, 8], defining these as key barriers across different settings. Lack of time is attributed to heavy clinical workload demand over academic work. Lack of time is a well-recognized barrier that is cited in medical literature. An effective literature search takes time and a recent study done in Australia has demonstrated that time pressure leads to high stress levels, ineffective searches and lower confidence in the answers [29]. Doctors busy with clinical duties may not have time to effectively search literature. Therefore, lack of time is an important impediment to the adoption of EBM and warrants effective measures to enhance the practice of EBM at AKUHN.

Two other common barriers faced by AKUHN residents were insufficient basic EBM skills (a personal barrier) and patients’ unawareness about EBM (patient-related barrier). Similarly, an Ethiopian study reported lack of skills as an important barrier [11]. Searching for literature and critical appraisal were perceived as the most difficult steps because searching takes time and there is lack of strong basic knowledge in statistics. Residents in this study felt that this challenge stems from the lack of training in critical thinking and problem solving skills during the early school years- they were ill equipped to “think outside the box” while in school. Recently the Kenyan government has introduced reforms in the education system to move away from the traditional teaching methods and put more emphasis on improving problem-solving skills [30]. In fact, in January 2019, Kenya initiated the nation-wide implementation of a Competency Based Curriculum (CBC) in primary schools, which includes teaching of communication skills, critical thinking and problem-solving [31]. However, a study conducted by institute of educational development has concluded that implementation of the new CBC programme is still a challenge due to lack of human resources, inadequate learning and teaching materials and lack of parental support for the new reform [32]. Early introduction of EBM right from medical school and teaching of critical thinking skills from primary and secondary school level would mitigate the barrier of poor critical thinking skills.

In studies done in Japan, Ethiopia and Sudan, “patients’ unawareness about EBM” was a significant barrier to EBM practice [5, 8, 11]. In this study, residents also rated patients’ unawareness about EBM as a significant barrier in the quantitative survey. On the other hand during the interviews, residents felt that patients visiting AKUHN were very “well-informed” and saw that as a facilitator of EBM. Most residents took that as an opportunity to read more and to “up their game”. Likewise, a survey among 1050 physicians in United States of America showed that a majority of the physicians believed that patients bringing information to the visit had a beneficial effect on the physician-patient relationship but this would worsen if patients seemed to be challenging physician’s authority or asking for inappropriate treatment [33].

## Conclusions

There was good understanding and support of EBM among residents at AKUHN, though evidence based practice remained a challenge.

In a setting where significant resources were spent to equip residents with the most current EBM resources and to provide training in EBM skills, the findings of relatively low practice and utilization of EBM resources are revealing. This means making EBM resources available to residents is only one of the facilitating factors in ensuring that EBM is practiced routinely and with ease. These study findings are useful to planners and educators in this and other institutions.

The major recommendation from this study is that enhancing the use of EBM so that residents can increase their facility with and use of EBM in their clinical care of patients is advisable, but resources should be allocated wisely. Expenditure on institutional EBM resources and training does not necessarily translate into practice and utilization of EBM. Perhaps, more efforts need to be directed towards cultivating critical thinking skills at an early stage in physicians’ careers by integrating EBM into undergraduate medical curricula. In this way, students will have an easier transition into residency and will have gained more confidence in EBM skills.

## Strengths and limitations

The high response rate of the study gave us a large sample and higher statistical power to obtain statistically significant effects. The high response rate also eliminated the risk of non-response bias. Additionally, the mixed method nature of this study allowed triangulation of quantitative and qualitative findings.

The major limitation of this study was that it relied on residents’ self-assessment of their knowledge and attitudes. They may have responded inaccurately in order to be viewed in a positive light by the researcher. However this risk of social desirability bias was mitigated by anonymizing the survey and by repeatedly reassuring the participants of the same during the IDIs.

## Supplementary Information

**Additional file 1.** Questionnaire on the Self-reported knowledge, attitudes, practices and barriers of EBM among resident physicians at AKUHN (DOCX 112.4 KB)

**Additional file 2.** An interview guide for the qualitative part of the study on the “Self-reported knowledge, attitudes, practices and barriers of EBM among resident physicians at AKUHN” (DOCX 97.2 KB)

**Additional file 3.** EBM Quantitative dataset (XLSX 47.0 KB)

## Data Availability

The quantitative data generated and analyzed during this study are included in the supplementary files (Additional file 3). Transcripts from qualitative study will not be shared to protect the anonymity of the residents.

## Abbreviations

AKUHN: Aga Khan University Hospital Nairobi
CBC: Competency-Based Curriculum
CI: Confidence Interval
EBM: Evidence Based Medicine
IDIs: In-depth Interviews
LMIC: Low and Middle Income Countries
MMed: Master of Medicine
PGME: Post Graduate Medical Education
SD: Standard Deviation
SSA: Sub Saharan Africa
