# Supplementary material for: Self-reported knowledge, attitudes, practices and barriers in use of evidence-based medicine among resident physicians in Kenya: a mixed methods study
Source: BMC Med Educ. 2021 Oct 23;21:537. doi: 10.1186/s12909-021-02974-4 (PMC8542330; doi:10.1186/s12909-021-02974-4)
Supplement: Supplementary file 1 — Additional file 1. Questionnaire on the Self-reported knowledge, attitudes, practices and barriers of EBM among resident physicians at AKUHN (DOCX 112.4 KB) [file 12909_2021_2974_MOESM1_ESM.docx]

**Additional file 1: Questionnaire**

**Questionnaire on the self-reported knowledge, attitudes, practices and barriers of EBM among resident physicians at AKUHN**

| **SECTION 1: SOCIO-DEMOGRAPHIC CHARACTERISTICS** | | | | | | | | | | | | |
| --- | --- | --- | --- | --- | --- | --- | --- | --- | --- | --- | --- | --- |
| No | Question | | | | Response | | | | | | Code | |
| 1 | Sex | | | | 1. Male 2. Female | | | | | | [ ] | |
| 2 | Age (years) | | | |  | | | | | | [ ] | |
| 3 | Department enrolled | | | | 1. General Surgery 2. OB/GYN 3. Internal Medicine 4. Paediatrics & Child Health 5. Anaesthesiology 6. Radiology 7. Family Medicine 8. Anatomic Pathology 9. Clinical Pathology | | | | | | [ ] | |
| 4 | Year of residency | | | | 1. One 2. Two 3. Three 4. Four | | | | | | [ ] | |
| **SECTION 2: KNOWLEDGE ABOUT EBM** | | | | | | | | | | | | |
| 5 | Below is a list of EBM resources most used by resident physicians. Please indicate those which you have used or are aware of. | | | | | | | | | | | |
|  | EBM resources | | Not aware | | Aware but never use | | Aware and use occasionally | | | Aware and use frequently | | Aware and use almost every time |
|  | UpToDate | |  | |  | |  | | |  | |  |
|  | Cochrane database of Systematic Reviews | |  | |  | |  | | |  | |  |
|  | PubMed/Medline | |  | |  | |  | | |  | |  |
|  | Hinari | |  | |  | |  | | |  | |  |
|  | ClinicalKey (Elsevier) | |  | |  | |  | | |  | |  |
|  | Medicine (McGraw Hill) | |  | |  | |  | | |  | |  |
|  | Google Scholar | |  | |  | |  | | |  | |  |
|  | BMJ Clinical Evidence | |  | |  | |  | | |  | |  |
|  | Others | |  | | | | | | | | | |
| 6 | This section of the questionnaire inquires about your knowledge of terminologies used in EBM. Below is a list of some of the common EBM terms used, please describe your level of understanding of each: | | | | | | | | | | | |
|  | Terminology used in EBM | Lack of knowledge about term | | | Little knowledge about term | | | Under-standing  the term | Understanding & using the term without defining it | | | Understanding & defining the term |
|  | Evidence-based practice (EBP) |  | | |  | | |  |  | | |  |
|  | Relative risk |  | | |  | | |  |  | | |  |
|  | Absolute risk |  | | |  | | |  |  | | |  |
|  | Odds ratio |  | | |  | | |  |  | | |  |
|  | Sensitivity & specificity |  | | |  | | |  |  | | |  |
|  | Confidence interval |  | | |  | | |  |  | | |  |
|  | Systematic review |  | | |  | | |  |  | | |  |
|  | Meta analysis |  | | |  | | |  |  | | |  |
|  | Bias |  | | |  | | |  |  | | |  |
|  | Medical Subject Headings (MeSH) |  | | |  | | |  |  | | |  |
| 7 | Among all the EBM skills listed below, please describe your level of confidence for each skill: | | | | | | | | | | | |
|  | EBM skills | Very poor | | | Poor | Acceptable | | | | Good | | Very Good |
|  | Formulating a clinical question |  | | |  |  | | | |  | |  |
|  | Searching the literature |  | | |  |  | | | |  | |  |
|  | Critical Appraisal of the literature |  | | |  |  | | | |  | |  |
|  | Extrapolating literature findings to the patient in question |  | | |  |  | | | |  | |  |
|  | Evaluation the effectiveness of the intervention |  | | |  |  | | | |  | |  |
| **SECTION 3: ATTITUDE TOWARDS EBM** | | | | | | | | | | | | |
| 8 | This section inquires about your preferences of information sources to guide your clinical decision-making. For the statements listed below, please mark an X on the response that most accurately reflects your preference(s): | | | | | | | | | | | |
|  |  | Never | | | Rarely | | Sometimes | | | Most times | | Every time |
|  | Consult senior directly |  | | |  | |  | | |  | |  |
|  | Consult colleagues directly |  | | |  | |  | | |  | |  |
|  | Consult clinical practice guidelines |  | | |  | |  | | |  | |  |
|  | Attend CME and present your case |  | | |  | |  | | |  | |  |
|  | Search for evidence using EBM resources |  | | |  | |  | | |  | |  |
|  | Read medical textbook |  | | |  | |  | | |  | |  |
|  | Use electronic search engine (Yahoo, Google, etc.) |  | | |  | |  | | |  | |  |
| 9 | This section inquires about your opinions about EBM. For the statements listed below, please mark with an X the response that most accurately reflects your current opinion about EBM. | | | | | | | | | | | |
|  |  | Strongly disagree | | | Disagree | | Not sure | | | Agree | | Strongly agree |
|  | EBM improves overall patient outcomes |  | | |  | |  | | |  | |  |
|  | EBM helps in clinical decision making |  | | |  | |  | | |  | |  |
|  | EBM practice reduces healthcare costs |  | | |  | |  | | |  | |  |
|  | EBM brings about quick knowledge update |  | | |  | |  | | |  | |  |
|  | EBM should be taught to undergraduate medical students |  | | |  | |  | | |  | |  |
|  | EBM is equal to research activity |  | | |  | |  | | |  | |  |
|  | EBM application is difficult in daily practice |  | | |  | |  | | |  | |  |
|  | EBM is suitable only for research based institutions |  | | |  | |  | | |  | |  |
|  | EBM has no relevance in low resource settings |  | | |  | |  | | |  | |  |
|  | EBM is a mantra with no direct applicability to patients in rural settings |  | | |  | |  | | |  | |  |
|  | The costs of EBM outweigh its benefits |  | | |  | |  | | |  | |  |
| **SECTION 4: PRACTICE OF EBM APPLICATION** | | | | | | | | | | | | |
| 10 | Below is a list of some of the most common reasons that resident physicians use EBM during clinical practice. For the statements listed below, please mark with an X the response that most accurately reflects your reason(s) for using EBM in your clinical practice. | | | | | | | | | | | |
|  | Reason for application of EBM | Never | | | Rarely | Sometimes | | | | Often | | Always |
|  | Patient not improving |  | | |  |  | | | |  | |  |
|  | On encountering an uncommon case |  | | |  |  | | | |  | |  |
|  | Treating a new patient |  | | |  |  | | | |  | |  |
|  | For grand rounds or case discussions |  | | |  |  | | | |  | |  |
|  | Others (Specify) |  | | | | | | | | | | |
| 11 | Based on the cases you have seen within the last month, for the statements listed below, please mark with an X the response that most accurately reflects your experience. | | | | | | | | | | | |
|  | Statement | None of the cases | | For less than half of the cases | | | | For half of the cases | | For more than half of the cases | | For all the cases |
|  | How much did you rely on your knowledge in order to provide the appropriate treatment? |  | |  | | | |  | |  | |  |
|  | How much did you rely on your consultants to provide the treatment? |  | |  | | | |  | |  | |  |
|  | How often in the last month did you use EBM resources to answer a clinical question? |  | |  | | | |  | |  | |  |
|  | How much did you rely on anecdotal evidence? |  | |  | | | |  | |  | |  |
|  | How often did you apply what you read in articles to your patients? |  | |  | | | |  | |  | |  |
| **SECTION 5: BARRIERS OF EBM APPLICATION** | | | | | | | | | | | | |
| 12 | This section inquires about the possible barriers to the practice of EBM at AKU. For the statements listed below, please mark with an X the response that most accurately reflects your experience. | | | | | | | | | | | |
|  | Barriers | Strongly  disagree | | | Disagree | | | Don’t know | | Agree | | Strongly Agree |
|  | Lack of familiarity with EBM |  | | |  | | |  | |  | |  |
|  | EBM practice devalues clinical experience |  | | |  | | |  | |  | |  |
|  | Impracticality of EBM for everyday use |  | | |  | | |  | |  | |  |
|  | EBM removes the ‘art’ of medicine |  | | |  | | |  | |  | |  |
|  | EBM de-emphasizes history taking and physical examination skills |  | | |  | | |  | |  | |  |
|  | Lack of time to access EBM sources |  | | |  | | |  | |  | |  |
|  | Lack of access to internet and EBM sources |  | | |  | | |  | |  | |  |
|  | Insufficiency of basic EBM skills |  | | |  | | |  | |  | |  |
|  | Skepticism over the quality of evidence |  | | |  | | |  | |  | |  |
|  | Patients’ unawareness about EBM and preference of traditional approach |  | | |  | | |  | |  | |  |
|  | Others |  | | | | | | | | | | |
