# Supplementary material for: Self-reported knowledge, attitudes, practices and barriers in use of evidence-based medicine among resident physicians in Kenya: a mixed methods study
Source: BMC Med Educ. 2021 Oct 23;21:537. doi: 10.1186/s12909-021-02974-4 (PMC8542330; doi:10.1186/s12909-021-02974-4)
Supplement: Supplementary file 2 — Additional file 2. An interview guide for the qualitative part of the study on the “Self-reported knowledge, attitudes, practices and barriers of EBM among resident physicians at AKUHN” (DOCX 97.2 KB) [file 12909_2021_2974_MOESM2_ESM.docx]

## Additional file 2: Interview guide

**An interview guide for the qualitative part of the study on the “Self-reported attitudes, knowledge, practice and barriers of EBM among resident physicians at AKUHN”**

**A: PRE AMBLE (10 minutes)**

1. Self introduction
2. Refreshment
3. Briefings on purpose of this discussion
4. Distribute the participant information letter (Appendix C)
5. Clarify any queries
6. Obtain written consent for the interview and audio-recording (Appendix D)

**B: DISCUSSION PHASE (50 minutes)**

**Attitude towards EBM**

1. What is the first thing that comes to mind when you think about EBM?
2. It is said that evidence-based medicine improves patients’ care! What is your opinion?

- Why?

1. Do you think EBM is important for residents learning?

- If yes, tell me more about it?
- If no, why is that?

1. Do you think EBM should be taught in medical school?

- If yes, why?
- If no, why?

**Practice & Barriers of EBM**

1. It is said that consultants play a major role in influencing how residents practice medicine. What is your opinion?
   1. Do consultants keep abreast with the evidence? How?
   2. Probe for an example ….
2. From the literature, patients also play an important role in the way residents practice medicine. What do you think?

- Why?
- Literature shows some physicians intimated by informed patients. Is this true?
- Give an example of where a patient has encouraged/discouraged you….

1. Do you find it easy to search for clinical information while on duty at the hospital?

- If yes, what are the facilitators?
- If no, what are the barriers?
  - Workload – what can be done?

1. On a normal day in the clinic, do you generate clinical questions while you are seeing patients?
   - If no, why not?
   - If yes, on average, how many questions do you generate per 10 patients you see?
     1. How many of them do you look up?
     2. How do you select which question to pursue?

**Knowledge about EBM**

1. Where did you learn about EBM?
2. In the AKU orientation week, was the training useful?
   1. If yes, why?
   2. If not, why not and how can it be made better?
3. Do you think EBM should be taught in medical school?

- If yes, why?
- If no, why?

1. What aspects of EBM do you find most challenging?

- Why?

1. Do you think EBM can be taught?

- If yes, what teaching methods do you believe are most effective in teaching residents to apply EBM in their practice?
- What teaching methods do not work?

1. AKU library invests a lot in resources for the residents. Do you think it is useful?
   1. If yes, how?
   2. If not, how can it be made better?
2. How has AKU prepared/not prepared you to practice EBM after residency?

**Final remarks**

1. Any recommendations on how EBM can be improved in AKU?
2. How would you rate AKU as an institution in terms of its practice of EBM?
3. Finally, any final suggestions/comments about EBM in AKU?
